# Increased sHLA-G Is Associated with Improved COVID-19 Outcome and Reduced Neutrophil Adhesion

**DOI:** 10.3390/v13091855

**Published:** 2021-09-17

**Authors:** Daria Bortolotti, Valentina Gentili, Sabrina Rizzo, Giovanna Schiuma, Silvia Beltrami, Savino Spadaro, Giovanni Strazzabosco, Gianluca Campo, Edgardo D. Carosella, Alberto Papi, Roberta Rizzo, Marco Contoli

**Affiliations:** 1 Department of Chemical, Pharmaceutical and Agricultural Science, University of Ferrara, 44121 Ferrara, Italy; daria.bortolotti@unife.it (D.B.); valentina.gentili@unife.it (V.G.); sabrina.rizzo@unife.it (S.R.); giovanna.schiuma@unife.it (G.S.); silvia.beltrami@unife.it (S.B.); giovanni.strazzabosco@unife.it (G.S.); 2 Intensive Care Unit, Department of Translational Medicine, University of Ferrara, 44121 Ferrara, Italy; savino.spadaro@unife.it; 3 Cardiology Unit, Azienda Ospedaliero Universitaria di Ferrara, Cona, 44124 Ferrara, Italy; gianluca.campo@unife.it; 4 CEA, Institute of Emerging Diseases and Innovative Therapies (iMETI), Research Division in Hematology and Immunology (SRHI), Saint-Louis Hospital, 75001 Paris, France; edgardo.carosella@cea.fr; 5 Respiratory Section, Department of Translational Medicine, University of Ferrara, 44121 Ferrara, Italy; alberto.papi@unife.it (A.P.); marco.contoli@unife.it (M.C.); 6 Respiratory Unit, Azienda Ospedaliera Universitaria Ferrara, Cona, 44124 Ferrara, Italy; 7 Industrial Research and Technology Transfer Laboratory (LTTA), University of Ferrara, 44121 Ferrara, Italy

**Keywords:** HLA-G, COVID-19, E-selectin, ICAM-1, CD160, neutrophil

## Abstract

Human leukocyte antigen (HLA) is a group of molecules involved in inflammatory and infective responses. We evaluated blood sHLA-E and sHLA-G levels in hospitalized COVID-19 patients with respiratory failure and their relationship with clinical evolution, changes in endothelial activation biomarker profile, and neutrophil adhesion. sHLA-E, sHLA-G, and endothelial activation biomarkers were quantified by ELISA assay in plasma samples. Neutrophil adhesion to endothelium was assessed in the presence/absence of patients’ plasma samples. At admission, plasma levels of sHLA-G and sHLA-E were significantly higher in COVID-19 patients with respiratory failure compared to controls. COVID-19 clinical improvement was associated with increased sHLA-G plasma levels. In COVID-19, but not in control patients, an inverse correlation was found between serum sICAM-1 and E-selectin levels and plasma sHLA-G values. The in vitro analysis of activated endothelial cells confirmed the ability of HLA-G molecules to control sICAM-1 and sE-selectin expression via CD160 interaction and FGF2 induction and consequently neutrophil adhesion. We suggest a potential role for sHLA-G in improving COVID-19 patients’ clinical condition related to the control of neutrophil adhesion to activated endothelium.

## 1. Introduction

Human leukocyte antigen (HLA)-E and HLA-G belong to ‘non-classical’ HLA-class Ib molecules, which also includes -F and -H [1,2]. In contrast with highly polymorphic HLA-class Ia molecules (HLA-A, -B, and -C), HLA-Ib molecules display a low degree of polymorphism and different immunoregulatory properties [3]. Several results supported a correlation between HLA-E and HLA-G expression in physiological and pathological conditions [4,5]. HLA-G molecules interact with immune inhibitory receptors (ILT2, ILT4, KIR2DL4), modulating the functions of NK (Natural Killer) cells, T cells, B cells, and [6,7]. Thus, HLA-G molecules are involved in the control of infective and inflammatory conditions [8]. Moreover, HLA-G interact with endothelial cells via the CD160 receptor [9], a glycosylphosphatidylinositol-anchored member of the immunoglobulin superfamily. The interaction between soluble (s)HLA-G and CD160 on the surface of endothelial cells induces the apoptosis of endothelial cells, inhibiting the angiogenetic process via down-regulation of fibroblast growth factor 2 (FGF2) [9]. The expression of HLA-G molecules is partly controlled by genetic background, with HLA-G*0105N alleles presenting significantly reduced sHLA-G expression levels in comparison with HLA-G*0101 alleles [10].

HLA-E expression on the cell surface needs interaction with nonapeptides derived from leader peptides from HLA-I molecules and beta2 microglobulin [11]. HLA-E interacts with the NKG2A/CD94 inhibitory receptor, exerting immunosuppressive functions on NK cell and CD8+ T cell-mediated lysis [12,13]. Recently, the increased expression of soluble (s)HLA-E, generated by metalloproteases-dependent shedding of the membrane-bound molecule, has been observed in pathological conditions, such as multiple sclerosis, melanoma, and juvenile idiopathic arthritis [14]. sHLA-E is secreted by activated endothelial cells [15,16] and the levels might be controlled by genetic background, with a higher expression in the HLA-E*0103 allele in comparison with the HLA-E*0101 allele [17]. Recently, it has been suggested that there is a role for these molecules in the SARS-COV2 infection [18], supporting our data on the expression of HLA-G molecules by epithelial cells of the intestinal mucosa and in some lymphocytes, in correspondence with SARS-COV2-positive sites [19].

The coronavirus disease 2019 (COVID-19) is a global public health issue. Approximately 170 million cases have been globally confirmed so far, with 3.5 million deaths (https://covid19.who.int; accessed on 5 August 2021) [20]. COVID-19 is an heterogenous disease associated with SARS-COV2 infection with a range of severity spanning from paucisymptomatic manifestations characterized by fever, cough, dyspnea, anosmia, ageusia, and fatigue up to respiratory distress syndrome (ARDS) and multiple organ failure with poor prognosis. Emerging evidence suggests that endothelial activation plays a central role in the pathogenesis of ARDS and multi-organ failure in patients with COVID-19. However, the molecular mechanisms underlying endothelial activation in COVID-19 patients remain unclear. Both HLA-E and HLA-G are involved in endothelial cells remodeling. HLA-G/CD160-mediated antiangiogenic property may participate in the vascular remodeling [9]. HLA-E expression and release of sHLA-E are features of endothelial cells activation and emphasize immunoregulatory functions of the endothelium [16]. The endothelial cells response to immune stimulation consists of the up-modulation of molecules such as E-selectin and ICAM-1 (Intercellular Adhesion Molecule 1). E-selectin and ICAM-1 are probably the most specific, inducible endothelial cell- surface molecules which are involved in the adhesion of neutrophils to inflammatory endothelium [21]. On the contrary, HLA-G is a potent inhibitor of neutrophil adhesion to endothelial cells [15]. The modification in the expression of these molecules on the surface of endothelial cells might affect the adhesion of neutrophils during the COVID-19 inflammatory cascade. The increased infiltration of immature and/or dysfunctional neutrophil contributes to the imbalance of the lungs’ immune response and has been observed in severe COVID-19 cases [22,23].

Understanding the immune–inflammatory mechanisms that pave the way to disease manifestations can identify potential targets for pharmacological interventions. Here we evaluated blood sHLA-E and sHLA-G in hospitalized COVID-19 patients with respiratory failure in relation with the evolution of the clinical conditions and in relation with endothelial activation biomarker profile variations and neutrophil cells/endothelium interaction. In parallel, blood sHLA-G and sHLA-E were assessed and compared in a group of control hospitalized subjects with respiratory failure and healthy controls not associated with SARS-COV2 infection.

## 2. Materials and Methods

### 2.1. Patients

The study was an investigator-initiated, prospective, single-center study recruiting consecutive patients admitted to the Respiratory and Intensive Care Units of the Azienda Ospedaliera Universitaria di Ferrara (Ferrara, Italy) (Supplementary Material File S1). The study aimed to prospectively evaluate the pro-thrombotic status and systemic inflammatory biomarkers in moderate-to-severe COVID-19 patients and to correlate these biomarkers with clinical outcomes (Clinicaltrials.gov identifier NCT04343053). The design of the study has been described in detail in previous reports [24,25,26]. Briefly, patients were included if they had SARS-COV2 infection (confirmed by PCR-positive nasopharyngeal swab specimens) and respiratory failure (defined as arterial oxygen tension of <8.0 kPa (60 mmHg) at room air and oxygen saturation < 90%). All the patients were infected by the SARS-COV2 isolate clustered in the B1 clade, which included most of the Italian sequences during the enrollment period. Patients were recruited from April 1 until the end of May 2020. After enrollment (T1; Baseline), COVID-19 patients were assessed every 7 ± 2 days for an additional 2 consecutive visits (T2 and T3). A detailed description of study procedures has been previously published [24,25,26] and is also available in the online supplement. Two control groups were recruited: a group of patients hospitalized in the same period at the same hospital with acute respiratory failure due to respiratory/cardiovascular acute conditions and not related to SARS-COV2; a group of healthy controls. For plasma preparation, the blood samples were collected using 6 mL EDTA-containing tubes. The tubes were centrifuged for 15 min at 2200 rpm. All blood specimens were processed immediately for plasma collection and aliquots were stored at −80 °C.

### 2.2. sHLA-G Specific ELISA

As described previously [27,28], serum levels of sHLA-G (sHLA-G1/HLA-G5) were measured by enzyme-linked immunosorbent assay (ELISA) using monoclonal antibodies MEM-G/9 (Exbio; Vestec, CZ) as capture antibodies, respectively. The intra-assay coefficient of variations (CV), the inter-assay CV, and the limit of sensitivity were 1.4%, 4%, and 1 ng/mL.

### 2.3. sHLA-E Specific ELISA

ELISA for soluble HLA-E were performed as previously described [28]. Briefly, MaxiSorp Nunc-Immuno 96 microwell plates (Nunc A/S; Roskilde, Denmark) were coated overnight at 4 °C with 3D12 mAb, specific for HLA-E HC (eBioscience; Science Center Drive, San Diego, CA, USA). After three washes with PBS 0.05% Tween 20 (washing buffer), plates were saturated with 200 μL/w of PBS 2% BSA (Sigma; St. Louis, MO, USA) for 30 min at RT.100 μL of test samples (plasma) or standard (serial dilutions of total extract from normal peripheral blood mononuclear cells) were added to each well and incubated at RT for 2 h. After three washes, 100 μL of detection reagent (HRP-conjugated anti-beta2 microglobulin mAb, Exbio; Vestec, CZ) was added, and plates were incubated for 2 h at RT. After three washes, 100 μL of TMB (substrate for HRP) was added, and the reaction was stopped after approximately 10′ by adding H2SO4 5N. Absorbance at 450 nm was measured using Infinite 200 PRO spectrometer (Tecan Group Ltd.; Seestrasse, Männedorf, Switzerland). Results are expressed as arbitrary units/mL (1 unit = quantity of sHLA-E in 1 µg of total extract).

### 2.4. HLA-E Allele Assignment

Genomic DNA was extracted from peripheral blood samples using the TIANamp Blood DNA Kit (Tiangen Biotech; Beijing, China). Allele-specific quantitative real-time PCR (qRT-PCR) with two forward primers, respectively, was used to discriminate HLA-E*0101 and HLA-E*0103 alleles: E*0101F (5′-GCG-AGC-TGG-GGC-CCG-CCA-3′) and E*0103F (5′-GCG-AGC-TGG-GGC-CCG-CCG-3′). Each of the forward primers was combined with a common HLA-E-specific reverse primer: 5′-CCG-CCT-CAG-AGG-CAT-CAT-TTG-3′. Two PCR reactions for each sample were carried out in a 10 μL reaction solution containing 20 ng genomic DNA, 0.2 μmol/L allele-specific forward primer, 0.2 μmol/L common reverse primer, and 5 μL 2 × SYBR Premix Ex Taq (TaKaRa; Shiga, Japan). The PCR amplification was carried out at 95 °C for 10 min, 40 cycles at 95 °C for 15 s, and 65 °C for 40 s, followed by a final stage of product dissociation cycle, using Mastercycler ep realplex (Eppendorf; Hamburg, Germany). Allele discrimination was manually performed according to the different PCR amplification efficiencies for different alleles, which can be shown by the cycle of threshold (Ct) [17].

### 2.5. HLA-G Allele Assignment

HLA-G allele assignment was carried out using polymerase chain reaction (PCR) followed by sequencing analysis. Briefly exon 2, 3, and intron 2 were amplified with a primer pair: forward 5′- GGCTGA GAG GTC TAC AGG AGA T-3′ and reverse 5′-GCT CCC ACT CCA TGA GGT ATT-3′ and amplification of exon 4 was performed using the primers: forward 5′-GTA TCT GGT TCA TTC TTA GGA TGG-3′ and reverse 5′-AAG ACT GCT CTG GGA AAG G-3′. PCR product of exon 2, 3, and intron 2 was 822 bp, and 502 bp for exon 4. The polymerase chain reaction (PCR) program for exon 2, 3, and intron 2 was: after 95 °C for 10 min, 30 cycles of 94 °C for 1 min, and 60 °C for 45 s followed by 72 °C for 45 s, and for exon 4 was: after 95 °C for 5 min, 30 cycles of 94 °C for 1 min and 59 °C for 45 s followed by 72 °C for 45 s [29]. The products were then sequenced with capillary sequencing using the Applied Biosystems 3500 XL sequencer (ThermoFisher Scientific; Milan; Italy) and ‘plink’ software was used (http://pngu.mgh.harvard.edu/~purcell/plink/index.shtml; accessed on 10 September 2021) for allelic haplotyping.

### 2.6. Endothelial Activation Biomarkers Levels Assay

Plasma samples were analyzed for Angiopoietin-2, Endoglin, Endothelin-1, IL-33, vWF, s-RAGE, sICAM-1, P-SELECTIN, sVCAM-1, PAI-1, sE-Selectin, Tissue Factor, Thrombomodulin, sCD40L by Multipore multiplex immunoassay-based (Merck; Milan, Italy) using Luminex instrument (Luminex; Austin, TX, USA).

### 2.7. Cell Cultures

HUVEC were obtained by collagenase treatment of the umbilical vein as described previously [30]. The cells were cultured in fibronectin-coated tissue culture flasks (Costar; Cambridge, MA; USA) in RPMI-1640 (Gibco, Paisley, UK), supplemented with 10% heat-inactivated human serum and 10% bovine calf serum (BCS), 50 pg/mL heparin, 30 pg/mL endothelial growth factor (Collaborative Research Incorporated; Bedford, MA; USA), and antibiotics. HUVEC of passage 3 or 4 were grown to confluence in 75 cm 2 (approximately 3 × 10^6^ cells/flask) cell culture flasks (Costar) or in 24 macro wells (Costar) and activated with TNF-alpha (0.625–5 ng/mL) for 4 h [31]. Anti-E-selectin (CD62E; Diaclone; Besançon cedex; France) (20 ng/mL), anti ICAM-1 (EP14442Y; Abcam; Milan, Italy) (20 ng/mL), anti-HLA-G (Exbio; Vestec, CZ) (10 ng/mL), and anti-CD160 (EPR23644-24; Abcam; Milan, Italy) (20 ng/mL) azide-free antibodies were added to cell cultures for 24 h. An anti-dinitrophenyl hapten mAb (Rat IgG1, DakoCytomation, Denmark) (10 ng/mL) was used as a negative control [32]. PD 166866 FGF2 inhibitor (Santa Cruz; Dallas, TX, USA) (50 nM) was used to inhibit FGF2 in endothelial cells. Each treatment was maintained for 24 h.

### 2.8. Neutrophil Binding Assay

Neutrophils were isolated from peripheral blood of COVID-19-negative control subjects by Polymorphprep (Progen; Heidelberg, Germany) [33]. Isolated neutrophil was stained with BioTracker 488 Green Nuclear Dye (Sigma-Aldrich; Milan, Italy) and added to HUVEC culture (6 × 10^5^). Cells were incubated for 20 min in a 37 °C, 5% CO_2_ incubator. PRE-wash total neutrophil fluorescence was measured by fluorescence intensity of each well with an excitation wavelength of 500 nm and an emission wavelength of 515 nm (fluorescein filter set) in a FLUOstar spectrophotometer (BMG Labtech; ThermoFisher; Milan, Italy). The co-cultures were washed and the POST-wash total neutrophil fluorescence was determined. The percent adherence per well was determined.

### 2.9. sE-Selectin and sICAM1 Assay

Levels of E-selectin and sICAM-1 in cell culture supernatants were detected using the Human sE-Selectin/CD62E Quantikine and sICAM-1/CD54 ELISA Kit (ReDsystems; Minneapolis, MN, USA) according to manufacturer’s instructions and using a Multiskan Ascent ELISA plate reader (ThermoFischer Scientific; Milan, Italy).

### 2.10. FGF2 Expression Assay

FGF2 gene expression was assessed by ready-to-use assay (Hs00266645_m1; Applied Biosystems, ThermoFischer Scientific; Milan, Italy) following manufacturer instructions [34].

### 2.11. Statistical Analysis

The normality of each variable was checked by using the Kolmogorov-Smirnov test. Statistical analysis was performed by a parametric approach for several variables with normal distribution. Fisher’s exact test was used to compare allelic frequencies. Student’s *t*-test was used to compare plasma mean levels of sHLA-E, sHLA-G, sE-selectin, and sICAM-1 among the various groups. The Spearman rank correlation coefficient test was used to identify possible relationships among different variables. A value of *p* < 0.05 was accepted as statistically significant. The statistical analysis was performed by GraphPad software version 9.

## 3. Results

### 3.1. Study Population

Patients’ characteristics were previously reported [24,25,26]. We enrolled fifty-four COVID-19 patients, 11 control patients that presented respiratory failure [24,25,26], and 100 healthy control subjects. The hospitalization of the control group was necessary for cardiovascular (heart failure) or respiratory (pulmonary infiltrates/pneumonia) problems. The three groups of patients were matched for gender, age, BMI, and smoking history. The hospitalized patients were matched for number of comorbidities per patient, need of respiratory support at recruitment, and clinical improvement (Table 1). At baseline, no differences were found in blood cell counts (Table 2) between the two groups of patients, while healthy controls showed lower total blood leukocytes and neutrophils (Table 2). We did not find any difference for treatments between patients who died compared to patients who survived during the study follow-up [24]. At variance with COVID-19 patients, none of the patients in the control group received antiviral treatments or hydroxychloroquine.

### 3.2. Immunological Parameters Evaluation

At baseline (T1), we found higher blood levels of both sHLA-G and sHLA-E in COVID-19 patients compared to controls with respiratory failure (sHLA-G: Median (IQR) 11 (49.54) vs. 54 (165.87) ng/mL *p* < 0.01; sHLA-E: 11 (224.63) vs. 54 (672.22) ng/mL *p* < 0.001) and healthy controls (sHLA-G: Median (IQR) 100 (20.51) vs. 54 (165.87) ng/mL *p* < 0.001; sHLA-E: 100 (10.23) vs. 54 (672.22) ng/mL *p* < 0.001) (Table 2). Similarly, controls with respiratory failure presented higher levels of sHLA-G and sHLA-E in comparison with healthy controls (sHLA-G: Median (IQR) 11 (49.54) vs. 100 (20.51) ng/mL *p* = 0.01; sHLA-E: 11 (224.63) vs. 100 (10.23) ng/mL *p* < 0.001) (Table 2).

**Figure 1:**
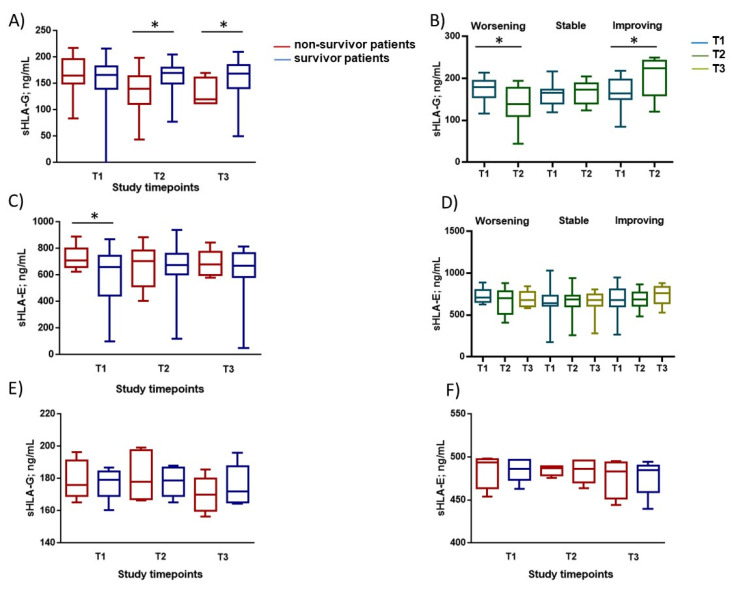
sHLA-G and sHLA-E concentration in blood of COVID-19 patients and controls. Blood sHLA-G and sHLA-E levels in COVID-19 patients and relationships with disease progression and clinical outcome: (**A**,**C**) Plasma sHLA-G and sHLA-E levels at baseline (T1) and at follow-up (T2 and T3; 7 ± 2 day interval between assessments) in non-survivor patients (red histograms) or survivor patients (blue histograms) during the study period. (**B**,**D**) Plasma sHLA-G and sHLA-E levels at baseline (T1) and after 7 ± 2 days (T2 and T3) on the basis of worsening, stability, or improving of the clinical manifestation of the disease (* *p* < 0.05, Student’s *t*-test). (**E**,**F**) Plasma sHLA-G and sHLA-E levels at baseline (T1) and at follow-up (T2 and T3; 7 ± 2 day interval between assessments) in non-survivor (red dots) or survivor (blue dots) control patients during the study period.

Although baseline levels of sHLA-G did not differ between survivors and non survivors for COVID-19 patients, the values significantly decreased over time in non-survivors (Figure 1A) (*p* = 0.036 at T2; *p* = 0.04 at T3). In control patients, sHLA-G levels decreased in both survivors and non-survivors over time (Figure 1E) with no statistical differences.

The increase of severity of COVID-19 from T1 to T2 (but not T2 to T3) was paralleled by a significant decrease of blood sHLA-G levels (Figure 1B) (*p* = 0.012; Student’s *t*-test). On the contrary, improved clinical conditions were paralleled by an increase in sHLA-G levels between T1 and T2 (*p* = 0.01; Student’s *t*-test).

Overall, blood sHLA-E was higher in non-survivors compared with survivors for COVID-19 patients (Figure 1C), (*p* = 0.016 at T1). The severity of the manifestation of COVID-19 did not affect the levels of sHLA-E (Figure 1D). In control patients, sHLA-E levels did not change over time in both survivors and non-survivors (Figure 1F).

### 3.3. Allelic Frequencies of HLA-G and HLA-E Genes

Since HLA-G and HLA-E molecules expression might be influenced by genetic background, we evaluated the distribution of *HLA-G* and *HLA-E* genes in the three groups. As reported in Table 3, we observed no differences in the allelic distribution between the three groups.

### 3.4. Correlations between Blood sHLA-G Levels and Endothelial Activation Biomarkers in COVID-19 Patients

**Figure 2:**
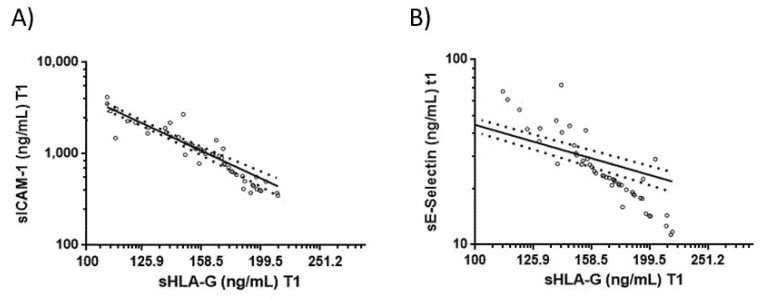
Correlations between sHLA-G concentration and sICAM and E-Selectin levels. Correlations between blood sHLA-G and (**A**) sICAM and (**B**) sE-Selectin levels in COVID-19 patients. Circles: single samples’ values; Line: fitted linear regression line; Dots: data distribution.

When all time points were considered, no correlations were found between sHLA-G and sHLA-E levels and blood inflammatory cell counts in COVID-19 patients and controls. Among the tested biomarkers, only E-selectin levels (r2 = 0.84; *p* < 0.0001, Figure 2A) and soluble intercellular adhesion molecule-1 (sICAM-1) levels (r2 = 0.83; *p* < 0.0001 Figure 2B) significantly and inversely correlated with blood baseline sHLA-G levels in COVID-19 patients. The tested biomarkers did not correlate with sHLA-G in control patients.

### 3.5. Endothelial Cell Response to HLA-G Molecules and Neutrophil Adhesion

**Figure 3:**
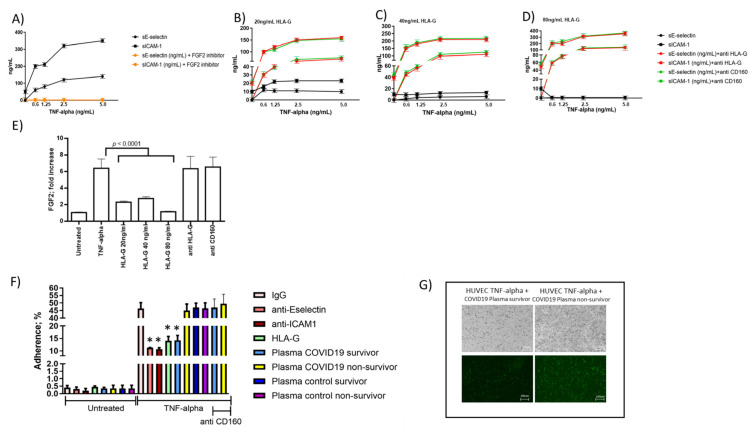
Endothelial cell in vitro assays. HUVEC endothelial cells were treated with TNF-alpha (0.625, 1.25, 2.5, 5.0 ng/mL) for 4 h and cultured overnight. (**A**) The levels of sICAM and sE-Selectin were assessed in culture supernatants in the absence or presence of FGF2 inhibitor. HUVEC endothelial cells were treated with TNF-alpha (0.625, 1.25, 2.5, 5.0 ng/mL) for 4 h and treated overnight with HLA-G molecules (20, 40, 80 ng/mL). (**B**–**D**) The levels of sICAM and sE-Selectin were assessed in culture supernatants in the absence or presence of anti-HLA-G (10 ng/mL) or anti-CD160 (20 ng/mL) antibodies. (**E**) Fold increase expression of FGF2 in TNF-alpha (2.5 ng/mL) activated HUVEC in the presence of HLA-G or anti-HLA-G, anti-CD160 antibodies in comparison with untreated HUVEC. (**F**) HUVEC endothelial cells were treated with TNF-alpha (2.5 ng/mL) for 4 h and cultured overnight in the presence of anti-E-Selectin (20 ng/mL), anti-ICAM1 (10 ng/mL), HLA-G molecule (40 ng/mL), and plasma samples (100 μL) from survivor and non-survivor patients in the absence (untreated) or presence of TNF-alpha treatment (2.5 ng/mL). The cells were then co-cultured with peripheral blood neutrophils from non-COVID-19 healthy controls and analyzed for neutrophil cells adhesion. anti-CD160 (10 ng/mL) was used to confirm the effect of interaction between plasmatic sHLA-G and CD160 in controlling neutrophil cells adhesion. A control IgG was used. * *p* < 0.05; Student’s *t*-test. (**G**) Images of BioTracker 488 Green Nuclear Dye-labeled neutrophils bound to untreated and TNFα-treated HUVEC monolayers incubated with plasma sample (100 μL) from survivor (left panel) and non-survivor (right panel) patients.

In COVID-19 patients, we observed an inverse correlation between baseline sHLA-G and the levels of these two adhesion molecules. Thus, as a proof of concept, we treated HUVEC cells with tumor necrosis factor alpha (TNF-alpha) and evaluated the levels of E-selectin and ICAM-1 molecules secretion in the presence or absence of HLA-G molecules. We observed an increase in both sE-selectin and sICAM-1 molecule secretion after TNF-alpha stimulation (Figure 3A) that decreased by the addition of HLA-G molecules (Figure 3B–D). The decrease in E-selectin and ICAM-1 molecules secretion was dose-dependent to HLA-G concentration. To evaluate the molecular mechanisms implicated in HLA-G control of E-selectin and ICAM-1 molecules secretion, we considered the HLA-G receptor CD160, expressed by endothelial cells [9]. HUVEC cells were treated with anti-HLA-G or anti-CD160 antibodies before HLA-G treatment. The addition of both anti-HLA-G and anti-CD160 antibodies restored E-selectin and ICAM-1 molecule secretion (Figure 3B–D). Since HLA-G/CD160 interaction is known to downregulate fibroblast growth factor 2 (FGF2) [9] and the expression of the endothelial cell adhesion molecules, as E-selectin and ICAM-1, are significantly up-regulated in the inflamed tissue by FGF2 [30], we evaluated its possible different expression. As a proof of the implication of FGF2 in E-selectin and ICAM-1 expression, we treated TNF-alpha-activated endothelial cells with a FGF2 inhibitor and we observed a reduction in both E-selectin and ICAM-1 induction (Figure 3A) (*p* < 0.0001; Student’s *t*-test). TNF-alpha treatment induced a significant increase in FGF2 expression which was reduced by HLA-G treatment (Figure 3E) (*p* < 0.0001; Student’s *t*-test). On the contrary, the pretreatment with anti-HLA-G or anti-CD160 antibodies reverted the decrease in FGF2 expression induced by HLA-G treatment (Figure 3E).

### 3.6. Correlations between Blood sHLA-G Levels and Neutrophil Adhesion to Activated Endothelial Cells

Since the modification of the expression of ICAM-1 and E-selectin might affect the adhesion of neutrophils during the inflammatory cascade [21], we tested the efficiency of neutrophil adhesion to endothelial cells in the presence of HLA-G molecules. We treated TNF-alpha activated endothelial cells with HLA-G molecules (40 ng/mL) and observed a reduction of the neutrophil adhesion to the endothelial cells comparable to the addition of anti-ICAM-1 and anti-E-selectin treatment (Figure 3F). Since the plasma samples from COVID-19 patients and controls differ for sHLA-G levels, we used them to treat endothelial cells. We observed that the plasma samples from survivor COVID-19 patients with the highest sHLA-G levels had a higher ability to inhibit neutrophil adhesion to endothelial cells, in comparison with COVID-19 non-survivor plasma samples (Figure 3F,G) (*p* < 0.001; Student’s *t*-test). Plasma samples from both survivor and non-survivor control patients behaved as COVID-19 non-survivor plasma samples, with no effect on neutrophil adhesion to endothelial cells (Figure 3F). The inhibition of neutrophil adhesion to endothelial cells obtained with plasma samples from survivor COVID-19 patients was significantly inhibited by anti-CD160 treatment (Figure 3F) (*p* < 0.001; Student’s *t*-test). Interestingly, the survivors’ plasma samples presented the highest levels of sHLA-G molecules in comparison with COVID-19 non-survivor plasma samples and control survivors and non-survivors.

## 4. Discussion

We report here for the first time the increased levels of sHLA-G and sHLA-E in plasma samples from hospitalized COVID-19 patients with respiratory failure.

We have demonstrated that sHLA-G and sHLA-E levels were higher in plasma samples from COVID-19 patients than in hospitalized control patients with respiratory failure at the time of admission and healthy controls. Hospitalized control patients with respiratory failure at the time of admission presented higher levels of sHLA-G and sHLA-E in comparison with healthy controls, showing the clinical condition as suggestive of an increase in the secretion of both molecules. Since the therapeutical treatment started after the admission, no confounding effect might be ascribed to therapeutical procedures. Moreover, no differences were found in terms of treatments between patients who died compared to patients who survived during the study follow-up. The evaluation of the genetic background did not show any differences in the three groups in terms of allelic frequencies. sHLA-G was increased in patients with improved clinical outcomes, thus suggesting that the increased concentration of sHLA-G in plasma samples may be related to inflammation and might reflect a peculiar feature of COVID-19 evolution. In fact, control patients showed a decrease in sHLA-G levels over time in both survivor and non-survivor patients. On the contrary, no correlation was found between serum sHLA-E levels and clinical outcomes of COVID-19 patients. HLA-G is known to have suppressive effects on the immune system [3]. Its deregulation has been implicated in both autoimmune and infectious diseases. In many autoimmune disorders, including celiac disease, rheumatoid arthritis, lupus, psoriasis, and diabetes, HLA-G upregulation is related to disease onset and progression [7]. Likewise, increased HLA-G levels have been found in infections of HIV-1, human cytomegalovirus, HPV, and herpes simplex virus-1, likely as a way to avoid immune detection of infected cells [7], and recently in patients with severe COVID-19 [35]. Thus, HLA-G upregulation might have a similar role in SARS-COV2 related immune dysfunction [36]. A case study by Zhang et al. reported the immune cell, cytokine, and HLA-G (including receptor) levels of a COVID-19 patient during hospitalization [37]. Overall, HLA-G levels decreased during the replication phase of COVID-19 and increased again after clearance, likely relating to corresponding cytokine levels. These data are in line with our findings of increased sHLA-G levels when clinical outcomes improve in COVID-19. We have previously reported that Natural Killer cells are affected by SARS-COV2 SP1 protein expression in lung epithelial cells via HLA-E/NKG2A interaction [38]. The resulting NK cells’ exhaustion might contribute to immunopathogenesis in SARS-COV2 infection.

As a proof of concept of the possible implication of sHLA-G levels in the COVID-19 course, we evaluated the levels of biomarkers of endothelial activation and correlated them with sHLA-G levels. Indeed, the endothelial compartment is a relevant target of SARS-COV2 infection which expresses ACE2 [39,40]. Endothelial dysfunction may play a major contribution to COVID-19 pathophysiology, leading to loss of physiological properties of the endothelium, including the ability to stimulate vasodilation, fibrinolysis, and anti-aggregation [41]. Previous studies found that endothelial dysfunction plays an important role in critical illness, especially in sepsis [42].

We observed an inverse correlation between sHLA-G levels and sICAM-1 and E-selectin levels in COVID-19 patients, but not in controls. We are aware that the main limitation of this research is the low number of control patients with respiratory failure. However, in the control group we recruited patients with similar presentation to COVID-19 patients admitted to hospital in the same clinical settings and over the same period of time. It is well known that during the first wave of the COVID-19 outbreak a significant reduction in hospitalization and admission for non-COVID-19 acute conditions occurred, leading to a limited number of patients suitable for the control group. However, we confirmed the ex vivo data in an in vitro setting. The in vitro analysis of activated endothelial cells confirmed the ability of HLA-G molecules to control sICAM-1 and sE-selectin expression via CD160 interaction and consequent FGF2 induction. Endothelial cells as well as leucocytes express adhesion molecules that are induced by transcription factors such as FGF2 [34], which mediate the adhesion and subsequent migration of leucocytes into tissue. De novo expression or enhanced expression of E-selectin and ICAM-1 has been described in inflammatory conditions [43,44,45]. The regulation of expression of these adhesion molecules is considered to play a major role in the localization and development of an inflammatory reaction. E-selectin and ICAM-1 are structurally unrelated adhesion molecules for granulocytes, monocytes, and T lymphocytes [46]. The inflammatory cytokines tumor necrosis factor (TNF), interleukin-1 (IL-I), interferon-y (IFN-y), and bacterial endotoxins (lipopolysaccharides (LPS)) alpha, and LPS are known inducers and enhancers of E-selectin and ICAM-1 [46]. Circulating leukocytes enter inflamed tissues through sequential adhesive and signaling events [47]. Neutrophils first tether to and roll on E-selectin expressed on activated endothelial cells, which enables interactions with ICAM-1 that promote arrest, adhesion strengthening, intraluminal crawling, and trans-endothelial migration. Importantly, E-selectin directly triggers signals in rolling neutrophils that cooperate with chemokine signals to maximize neutrophil recruitment during inflammation [48]. When we evaluated the effect of HLA-G molecules on neutrophil adhesion to activated endothelial cells, we observed that the addition of HLA-G molecules reduced the neutrophil adhesion to the endothelial cells with a comparable efficiency to that obtained with the addition of anti-ICAM-1 and anti-E-selectin treatment. As a proof of concept, we showed that the plasma samples from survivor COVID-19 patients had a higher ability to inhibit neutrophil adhesion to endothelial cells in comparison with COVID-19 non-survivor plasma samples. The inhibition of neutrophil adhesion to endothelial cells obtained with plasma samples from survivor COVID-19 patients was significantly inhibited by anti-CD160 treatment, suggesting a role of HLA-G/CD160 interaction in regulating neutrophil adhesion to endothelial cells. With less adhesion factors on endothelial cells, fewer neutrophils adhere to vessel walls and transmigrate into tissues, decreasing the overall detrimental effects observed in severe COVID-19 patients [49]. Although the role of HLA-G as an adhesion inhibitor has not been extensively studied, a study revealed the ability of HLA-G to block human natural killer rolling adhesion on porcine endothelial cells [50]. This is in agreement with our results on neutrophil cells and is of particular interest because the rolling adhesion of neutrophils represents the main mechanism of their recruitment to the injury site where they adhere tightly and migrate through the endothelium, causing inflammatory effects. The recognition of the possible mechanisms by which HLA-G might inhibit neutrophil adhesion to activated endothelial cells and may have significant anti-inflammatory properties [15]. Notably, the plasma levels of adhesion molecules, such as ICAM-1, fractalkine, vascular cell adhesion molecule-1 (VCAM-1), vascular adhesion protein-1 (VAP-1), and vascular endothelial growth factor (VEGF), have been reported to be elevated among COVID-19 patients, especially in severe cases [51]. These data, together with our results, suggest HLA-G and adhesion molecules are molecular mechanisms underlying COVID-19-induced endothelial injury, vascular permeability to neutrophil accumulation, angiogenesis, and pro-coagulation in COVID-19 pathogenesis.

**Figure 4:**
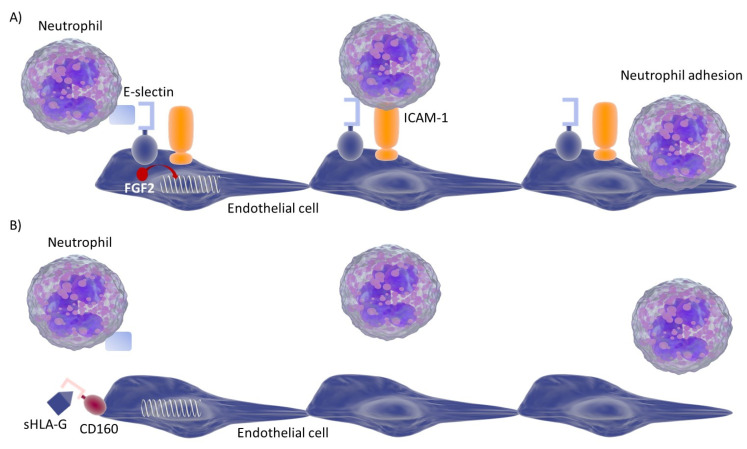
Representation of the molecular interaction on the basis of neutrophil cell adhesion to endothelial cells. (**A**) Neutrophils interact with E-selectin, enhancing ICAM-1 recognition and adhesion to endothelial cells. Both these molecules are induced by FGF2 (fibroblast growth factor 2). (**B**) In the presence of COVID-19 there is an increase in sHLA-G molecules interacting with CD160, which inhibits FGF2-dependent induction of E-selectin and ICAM-1. The reduction of E-selectin and ICAM-1 expression reduces neutrophil adhesion to endothelial cells. This condition might improve clinical conditions, reducing neutrophils activation.

In conclusion, our data suggest a potential role for sHLA-G in the control of neutrophil adhesion to activated endothelium in COVID-19 patients that is related to improvement of the disease (Figure 4). Thus, increased levels of sHLA-G in the blood may represent a novel, promising biomarker of disease activity in COVID-19. Further investigations are needed to assess HLA-G mechanisms to control ICAM-1 and E-selectin expression by activated endothelial cells and the neutrophil adhesion.

## Figures and Tables

**Table 1:** Demographic and clinical characteristics of the study population.

	Study Population (n = 165)				COVID-19 Patients (n = 54)		
	COVID-19 Patients, n = 54	Control Patients’ Respiratory Failure n = 11	Control Patients n = 100	*p*-Value *	Non-Survivor n = 16	Survivors n = 38	*p*-Value
Gender N (%)				>0.9			>0.9
Male	40 (74%)	8 (73%)	74 (74%)		12 (75%)	28 (74%)	
Female	14 (26%)	3 (27%)	26 (26%)		4 (25%)	10 (26%)	
Age	65 (57, 73)	70 (66, 76)	67 (56, 74)	0.2	72 (65, 78)	62 (55, 71)	0.004
Smoking habit N (%)							
Active smoker	0 (0)	3 (27%)	1 (1%)	0.003	0 (0)	0 (0)	NA
Former smoker	16 (30%)	4 (36%)	29 (29%)	0.725	7 (44%)	9 (24%)	0.2
BMI (kg/m^2^)	26.4 (24.2, 30.0)	24.8 (22.0, 27.1)	25.3 (23.1, 28.6)	0.13	28.5 (26.4, 30.9)	26.0 (24.1, 29.4)	0.2
Number of Comorbidities/patients	1.00 (0.00, 3.00)	2.00 (1.50, 3.00)	0 (0.00, 0,00)	0.12	3.00 (1.75, 4.00)	1.00 (0.00, 2.00)	0.004
Respiratory support at recruitment N (%)							
O_2_ only	11 (20%)	2 (18%)	NA		2 (12%)	9 (24%)	
HFNC or NIV	16 (30%)	6 (54%)	NA		4 (25%)	12 (31%)	
IV	27 (50%)	3 (27%)	NA		10 (62%)	17 (45%)	
Days from symptoms onset to recruitment	9 (5–14)	5 (2–8)	NA		10 (5–14)	8 (5–15)	0.60
Treatments N (%)							
Low molecular weight heparin	54 (100%)	11 (100%)	NA	>0.9	16 (100%)	38 (100%)	>0.9
Antibiotics	47 (87%)	10 (90%)	NA	>0.9	14 (88%)	33 (87%)	>0.9
Systemic corticosterods	37 (69%)	9 (81%)	NA	>0.9	12 (75%)	25 (66%)	0.7
Antivirals	29 (54%)	NA	NA	NA	7 (44%)	22 (58%)	0.5
Hydroxychloroquine	40 (74%)	NA	NA	NA	11 (69%)	29 (76%)	0.7

BMI, body mass index; HFNC, high flow nasal canula; NIV, non-invasive ventilation; IV, invasive ventilation. * *p*-value: COVID-19 patients vs. control patients’ respiratory failure.

**Table 2:** sHLA-G and sHLA-E levels and blood inflammatory cell counts at baseline (T1) in COVID-19 patients and controls.

	COVID-19 Patients, n = 54	Control Patients’ Respiratory Failure, n = 11	Control Patients, n = 100	*p*-Value *	*p*-Value **	*p*-Value ***
sHLA-G (ng/mL)	165.87 (44.3, 218.03)	49.54 (18.3, 54.9)	20.51 (0.0, 43.53)	0.01	<0.001	0.01
sHLA-E (ng/mL)	672.22 (173.9, 890.9)	224.63 (98.6, 310.4)	10.23 (0.0, 21.51)	0.001	<0.001	<0.001
Total blood leucocytes (cells × 10^3^/μL)	9.1 (6.8, 12.6)	12.0 (9.1, 14.7)	5.0 (4.1, 11.0)	0.2	0.023	0.021
Blood lymphocites (cells × 10^3^/μL)	0.83 (0.59, 1.04)	1.12 (0.52, 1.73)	0.96 (0.54, 1.29)	0.3	0.23	0.12
Blood Neutrophils (cells × 10^3^/μL)	7.9 (5.6, 10.2)	10.1 (5.8, 12.1)	3.2 (2.0–7.4)	0.2	0.01	0.01
Blood eosinophils (cells × 10^3^/μL)	0.04 (0.00, 0.14)	0.00 (0.00, 0.06)	0.00 (0.00, 0.02)	0.074	0.069	0.12

(Data are expressed as Median (IQR)). * *p*-value: COVID-19 patients vs. control patients’ respiratory failure. ** *p*-value: COVID-19 patients vs. control patients. *** *p*-value: Control patients vs. control patients’ respiratory failure.

**Table 3:** *HLA-G* and *HLA-E* allelic distribution in COVID-19 patients and controls.

	COVID-19 Patients n = 54	Control Patients’ Respiratory Failure n = 11	Control Patients n = 100	*p*-Value **	*p*-Value ***
HLA-E* alleles					
0101 N (%)	25 (47)	5 (46)	48 (48)	0.59	0.53
0103	28 (53)	6 (54)	52 (52)		
HLA-G* alleles					
0101 N (%)	47 (87)	9 (86)	85 (85)	0.63	0.76
0103	1 (1)	0 (0)	1 (1)		
0104	4 (8)	1 (7)	8 (8)		
0105N	2 (4)	1 (6)	6 (6)		

** *p*-value: COVID-19 patients vs. control patients’ respiratory failure. *** *p*-value: COVID-19 patients vs. Control patients.

## Data Availability

Data and statistical code are available from the corresponding author upon reasonable request.

## Supplementary Materials

The following are available online at https://www.mdpi.com/article/10.3390/v13091855/s1, File S1: Data collection.
