# Supplementary material for: Increased sHLA-G Is Associated with Improved COVID-19 Outcome and Reduced Neutrophil Adhesion
Source: Viruses. 2021 Sep 17;13(9):1855. doi: 10.3390/v13091855 (PMC8473385; doi:10.3390/v13091855)
Supplement: Supplementary file 1 [file viruses-13-01855-s001.zip › viruses-1360868-supplementary.pdf]

Increased sHLA-G is associated with improved COVID19 outcome and reduced neutrophil adhesion.

Running title: HLA-G and COVID19 clinical outcome

Daria Bortolotti[1], Valentina Gentili[1], Sabrina Rizzo[1], Giovanna Schiuma[1], Silvia Beltrami[1], Savino Spadaro[2], Gianluca Campo[3], Edgardo D. Carosella[4], Alberto Papi[5,6], Roberta Rizzo[1,7]*, Marco Contoli[5,6]*

*equally contributing

Data collection

An electronic case report form was used to collect anonymized demographic, clinical, laboratory and imaging data. Standard of care treatment, based on best available evidence, was used. After enrollment (T1—Baseline), COVID-19 patients were assessed every 7 ± 2 days for additional 2 consecutive visits (T2 and T3). At each time point, in line with the World Health Organization Ordinal Scale for Clinical Improvement (6), the level and changes of severity were identified on the basis of the type of intervention [3 steps scale: (1) oxygen supplementation; (2) high-flow oxygen or non-invasive mechanical ventilation (NIMV); (3) invasive mechanical ventilation (IMV)]. Furthermore, an increase of 50% or above of the ratio of arterial oxygen partial pressure (PaO2 in mmHg) to fractional inspired oxygen (FiO2 expressed as a fraction) (PaO2/FiO2 ratio) was considered a clinically relevant improvement. Study blood samplings were performed from an antecubital vein using a 21-gauge needle. All patients underwent blood sampling early in the morning before treatment administration. Baseline samples were obtained in all patients and COVID-19 patients were also sampled at each subsequent time-points. Blood samples were analyzed for inflammatory cell counts (performed at the central laboratory of the Azienda Ospedaliera Universitaria Ferrara, Italy).
